# Lamellar Hole-associated Epiretinal Proliferation in choroideremia: a case report

**DOI:** 10.1186/s40942-021-00333-5

**Published:** 2021-10-19

**Authors:** Vittoria Murro, Dario Pasquale Mucciolo, Dario Giorgio, Tomaso Caporossi, Ilaria Passerini, Daniele Bani, Fabrizio Giansanti, Gianni Virgili, Andrea Sodi

**Affiliations:** 1 Department of Neuroscience, Psychology, Drug Research and Child Health, University of Florence, Largo Brambilla, 3, 50134 Florence, Italy; 2 Ophthalmology Unit, San Jacopo Hospital, via Ciliegiole 97, 51100 Pistoia, Italy; 3 Department of Genetic Diagnosis, Careggi Teaching Hospital, Florence, Italy; 4 Department of Clinical and Experimental Medicine, Research Unit of Histology and Embryology, University of Florence, Florence, Italy; 5 Fondazione GB Bietti, Roma, Italy

**Keywords:** CHM, LHEP, Macular hole, Vitrectomy, Peeling, Electron microscopy, OCT, Lamellar Hole-associated Epiretinal Proliferation

## Abstract

**Background:**

To report a clinical case of a patient affected with choroideremia (CHM) who underwent macular surgery for a macular hole (MH) with Lamellar Hole-associated Epiretinal Proliferation (LHEP).

**Case presentation:**

We have described a 48-year-old male patient affected with CHM who developed MH with LHEP over a 7-year follow-up. The patient was referred to the Regional Center for Hereditary Retinal Degenerations of the Eye Clinic in Florence (Italy) in April 2012. The patient underwent vitrectomy and Inner Limiting Membrane (ILM) and LHEP peeling with fluid-air exchange. Ultra-structural examination of the excised epiretinal proliferation, carried out using electron microscopy, showed dense amorphous material, mainly composed of abundant clusters of fibrous collagens resembling compact fibrous long spacing collagen (FLSC), embedded in native vitreous collagen (NVC) and type IV collagen. No cells were detected in any of the specimens collected. At the 3rd-week postoperative follow-up the macular hole was closed.

**Conclusion:**

Macular hole with LHEP can be detected in CHM patients; in our patient the macular hole showed tractional and degenerative features, with good anatomical results after macular surgery.

## Background

Choroideremia (CHM) is a rare, X-linked, recessive disorder characterized by progressive degeneration of the outer retina, Retinal Pigment Epithelium (RPE) and choroid. It is caused by defects in the CHM gene (OMIM # 303100), on chromosome Xq21.2, which encodes Rab escort protein-1 (REP-1), a key mediator of membrane trafficking in the retina and RPE. Affected male patients develop night blindness in childhood and progressive constriction of the peripheral visual field begins in early adult life; usually only a very small island of central RPE and choroid with overlying fovea survives by the fifth decade of life. [1] In recent years few studies have investigated vitreoretinal abnormalities in patients affected with CHM. Macular changes associated with CHM include cystoid macular edema [2], choroidal neovascularization [3], epiretinal membrane and macular hole complicated by retinal detachment [4]. Although the development of a macular hole in CHM patients seems to be a rare event, recent works have highlighted favorable functional and morphological outcomes in symptomatic CHM patients undergoing pars plana vitrectomy for Full Thickness (FTMH) and Lamellar Macular Hole (LMH) [4–6]. In this study, we have described a patient affected with CHM who underwent macular surgery for a MH with Lamellar Hole-associated Epiretinal Proliferation (LHEP), developed during a 7-year follow-up; we focused on the morphologic features and ultra-structural findings of the LHEP in our CHM patient.

## Case presentation

**Fig. 1:**
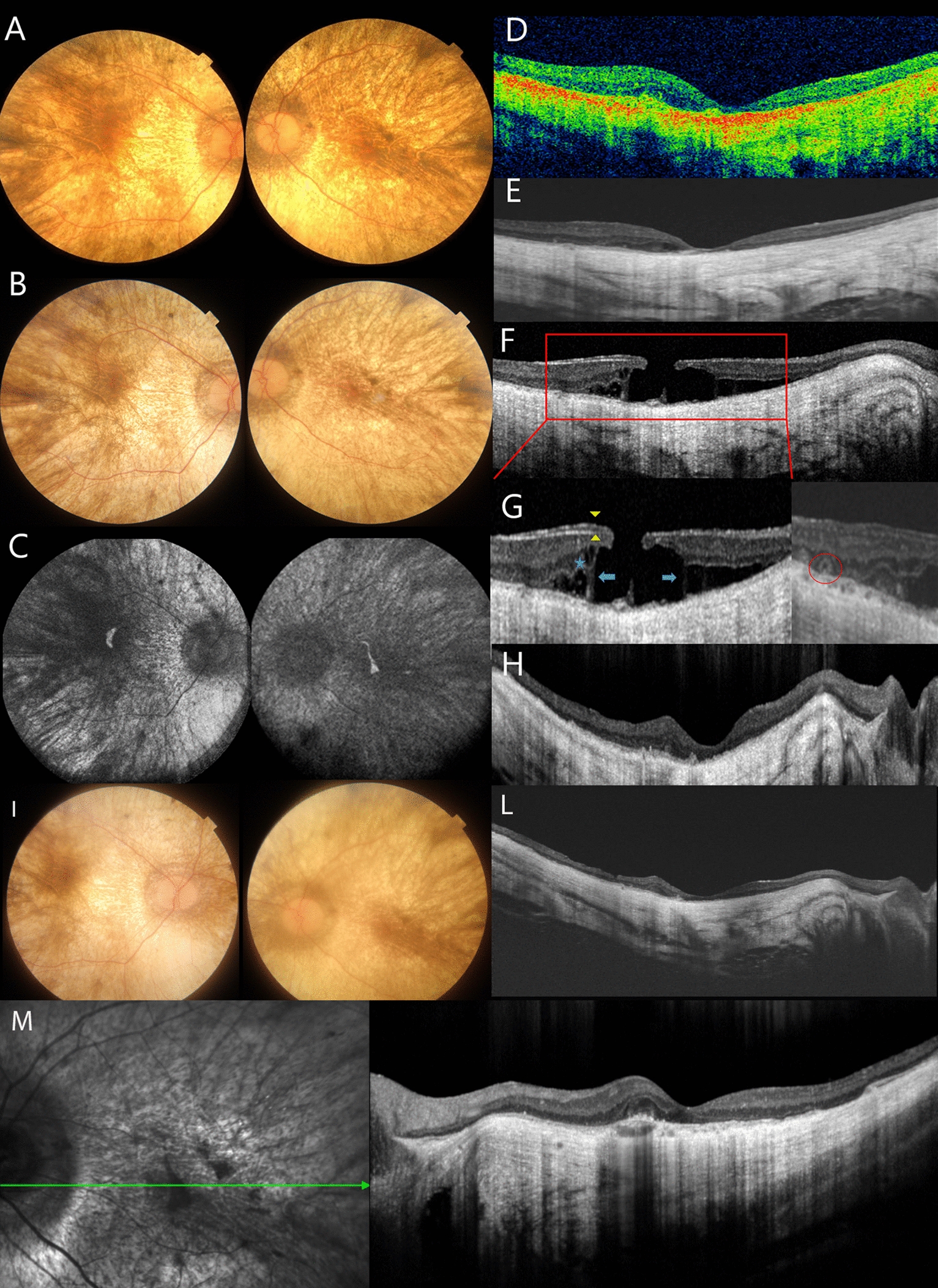
(**A**, **B**) Color fundus photographs (CFP) of the RE and LE respectively showing extensive chorioretinal atrophy at the referral in April 2012 (**A**) and in May 2017 (**B**). (**C**) Fundus autofluorescence (Blue-FAF) imaging showing an island of preserved RPE in the macula of both eyes. (**D**, **E**) OCT examination of the RE in 2012 (**D**) and in May 2017 (**E**) showing very thin neuroepithelium. (**F**) In October 2018, Epiretinal Proliferation (LHEP) is clearly visible attached to the inner surface of the retina. The red rectangle shows the magnified area in (**G**); the LHEP is outlined by yellow arrowheads. Two intraretinal cysts (blue asterisks) are visible at the inner nuclear layer and fine hyper-reflective bridges of tissue (blue arrows) across the neurosensory retina. Outer retinal tubulations (red circle) are detectable. (**H**) Post-surgical OCT scan shows closed macular hole (January 2019). (**I**) CFP of the RE and LE in July 2020; (**J**) OCT examination of the RE in July 2020. (**K**) Infrared-reflectance and OCT imaging of the left eye

**Fig. 2:**
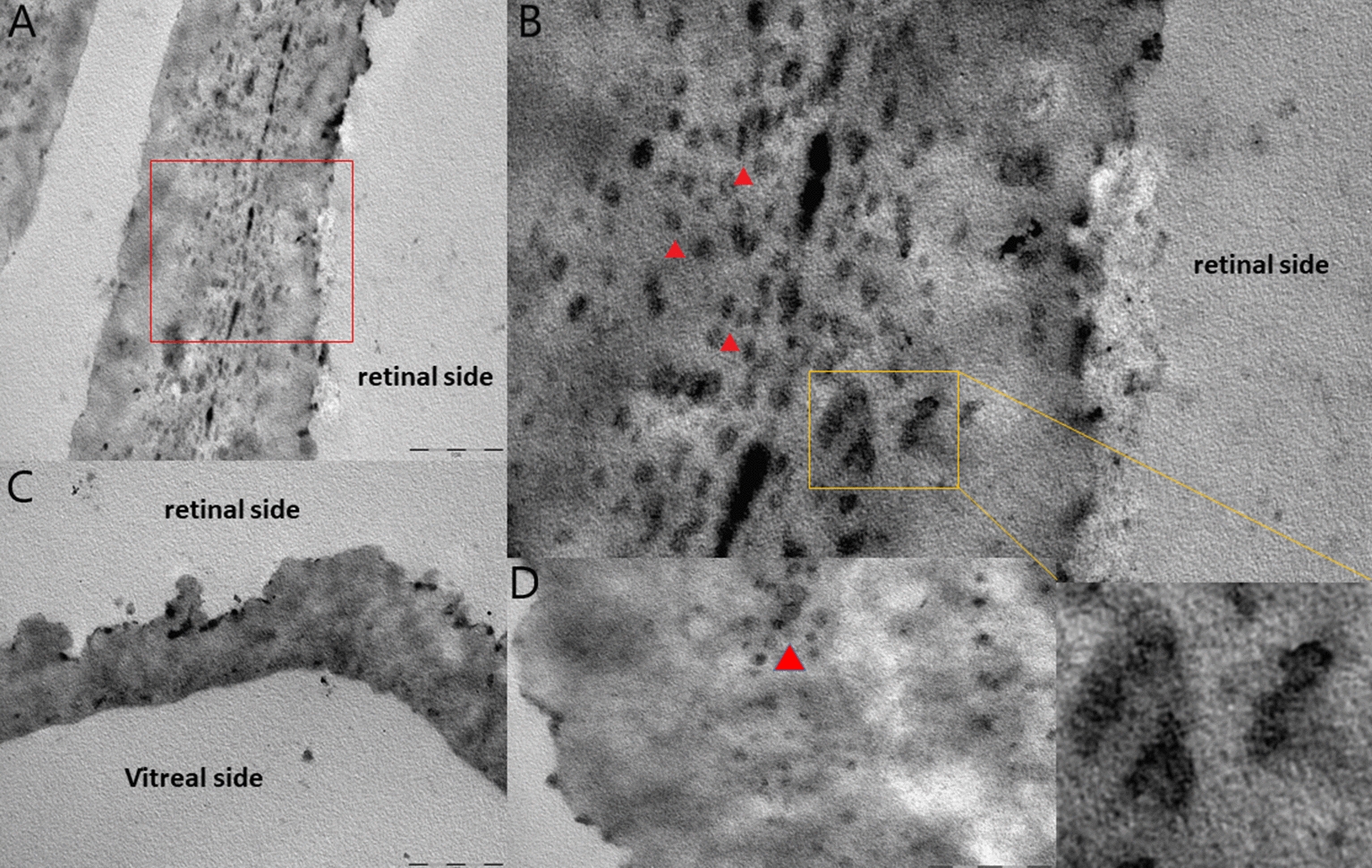
**A**–**D** Transmission electron microscopy images of epiretinal tissue. Image **A** showing dense extracellular matrix constituted of amorphous material of collagen strands (native vitreous collagen NVC and type IV collagen). The red square shows the magnified area in **B**. In Image **B** abundant clusters of fibrous collagen closely resembling compact FLSC (red arrowheads) embedded in native vitreous collagen (NVC) and type IV collagen are detectable. **C** The vitreal side of the epiretinal tissue appear smoother than the retinal side. **D** FLSC (detail in yellow square) embedded in vitreous collagen fibrils

A 48-year-old man was referred to the Eye Clinic of the University of Florence with bilateral decreased vision and hemeralopia in 2012. Best-Corrected Visual Acuity (BCVA) was 3.2/10 (0.50 LogMAR) in the Right Eye (RE) and 8/10 (0.10 LogMAR) in the Left Eye (LE). Spherical equivalent was − 6.0 and − 5.0 diopters in right and left eyes respectively. Slit lamp examination of the anterior segment showed mild posterior subcapsular lens opacities in the RE and clear lens in the LE. Intraocular pressure was normal. Fundoscopic examinations revealed a pale optic disc with widespread chorioretinal atrophy with a residual island of preserved retina at the macula of both eyes (Fig. 1A). Genetic examination performed at the Genetic Department of Careggi Teaching Hospital in Florence (Italy) identified the pathogenic deletion (c.1771−? _1962+? Del) on the *CHM* gene. During follow-up, the patient showed progressive worsening of vision in the RE. From January 2015 to May 2017, BCVA was stable (1/20; 1.30 LogMAR), while at the last visit (October 2018), BCVA was hand motion and 6.3/10 (0.20 LogMAR) in the right and left eyes respectively. OCT examination revealed the presence of a MH (which was not present at previous follow-ups). More specifically, avascular homogeneous material of medium reflectivity adherent to the inner retina at the margins of the macular hole resembling Lamellar Hole-associated Epiretinal Proliferation (LHEP) was detected (Fig. 1F, G). No hypo-reflective spaces were detectable between LHEP and the inner retina. The foveal edge appeared elevated. Schitic cavities, involving the residual atrophic neurosensory retina were characterized by hyper-reflective bridges of tissue crossing wider hypo-reflective spaces (typical of LMH) (Fig. 1G). Outer retinal tubulations were also present. Surgery was considered due to the decrease in visual acuity and the development of macular hole in the right eye. The patient underwent a standard 25-Gauge pars plana vitrectomy, complete removal of LHEP and peeling of the Internal Limiting Membrane (ILM) using Membrane Blue-Dual and fluid-air exchange. Simultaneous phacoemulsification was performed at the time of surgery. No complications were observed during surgery and one month later. At the 4-week postoperative follow-up the macular hole was closed (Fig. 1H); the visual acuity remained stable: hand motion in right eye. Pre- and post-operative microperimetry (MP-3, Nidek, Japan) examinations did not change and no improvement of the retinal sensitivity and fixation stability was observed post-surgery. The macular hole remained closed and visual acuity stable at the last follow-up (18 months after surgery, July 2020) (Fig. 1I, L). The epiretinal cell proliferation specimen was prepared and processed with standard fixation (Karnowski’s fluid and OsO4), embedding epoxy-resin, and sectioned for electron microscopy. Using transmission electron microscopy, the LHEP had a wavy retinal side and smooth vitreal side, and was made up of dense amorphous material mainly composed of abundant clusters of fibrous collagens, closely resembling compact fibrous long spacing collagen (FLSC) embedded in native vitreous collagen (NVC) and type IV collagen. NVC and collagen type IV were the 2 main types of collagen strands observed in the extracellular matrix (Fig. 2). In all the specimens collected neither fibroblasts, hyalocytes or myofibroblasts were identified. Written informed consent was obtained to report this clinical case.

## Discussion and conclusion

Several reports have described macular hole formation in chorioretinal dystrophies [7]. However, vitreoretinal abnormalities in CHM patients have been poorly investigated and macular hole development in CHM is very uncommon [5, 6]. Some authors have suggested vitreomacular traction as the causative mechanism of macular hole in CHM and concluded that the surgical closure of the macular hole is mandatory for patients who subsequently require subretinal gene therapy [5, 6].

In our case, we have reported the clinical course of a patient affected by CHM who developed a macular hole associated with extensive macular schisis and LHEP. To date, no previous works have reported on LHEP in CHM patients. More specifically we have reported the evolution of the macular changes during follow-up.

The presence of LHEP was shown to be related to the presence of photoreceptor layer defects and poor visual acuity, suggesting that eyes with a concurrent outer retinal pathology could have a higher propensity for LHEP formation [8–11].

Our CHM patient was characterized by severe outer retinal macular atrophy and the distinction between a lamellar macular hole and a full-thickness macular hole was difficult to identify due to the pre-existent outer retinal layer atrophy. However, the presence of hyper-reflective bridges of tissue crossing the entire retinal thickness suggested the onset of a lamellar macular hole; moreover, the presence of LHEP represents a criterion to identify a FTMH deriving from a LMH [12].

To date, lamellar macular holes are mainly classified in tractional (conventional) and degenerative (atypical) [13]. The first type is characterized by a hyperreflective line partially adherent to the ILM associated with tractional signs such as intra-retinal cysts and stretching of intra-retinal layers, with the outer retinal layer substantially preserved. The second type, also defined atypical ERM or “thick ERM”, is defined by a thicker homogenous band in contact with the underlying retina without evidence of traction; a disrupted ellipsoid band, round-edged cavitation and foveal bump are frequently associated with this type of hole [14]. However, a clear distinction cannot be achieved in all cases due to the presence of both tractional and degenerative features [11, 14–16]. Our clinical case showed the simultaneous presence of tractional and degenerative lamellar macular hole features. More probably, the onset of vitreomacular tractions led to the schitic abnormalities (tractional) in an almost completely atrophic neuroepithelium. The post-surgical results (closure of the hole and disappearance of schitic cavities) would confirm this hypothesis. Some authors have observed that gas reabsorption in CHM patients took approximately twice as long as would normally be expected with macular hole surgery, probably due to the extensive RPE and choroid atrophy [5, 6]; in fact, they recommended standard surgery using shorter-duration gas tamponade (SF6) in patients with CHM [5, 6]. For this reason, we preferred to use only air for our CHM patient. Furthermore, we have described morphologic characteristics of the LHEP using correlative microscopy. Although immunohistochemistry and electron microscopy have been widely used to understand the distribution and the composition of epiretinal tissue, most histological studies disagree about the origin of LHEP [17]. The ultrastructural composition of the excised ERM in our study (abundant cluster of FLSC in NVC) has already been reported in the LHEP of patients affected by idiopathic lamellar macular holes [18], but never reported in patients affected by CHM. Moreover, no cells were identified in any specimens analyzed, in contrast with previous works [18, 19]; this fact could be due to the advanced stage of the disease in our patient. Furthermore, we have to consider that it is difficult to obtain truly pure samples of LHEP due to attached hyaloid or concurrent tractional ERM; in addition, the LHEP specimen is microscopic, it is can be easily lost during the transfer of specimen from the eye to the laboratory.

In conclusion, we have described the clinical picture, surgical outcome, and ultrastructural features of a macular hole with Lamellar Hole-associated Epiretinal Proliferation in a CHM patient during a long-term follow-up.

## Data Availability

All data generated or analyzed during this study are included in this manuscript.
